# The Scientific Legacy of Bernard Maskit’s Work for Psychotherapy Practice and Research

**DOI:** 10.1007/s10936-025-10152-6

**Published:** 2025-05-03

**Authors:** Attà Negri

**Affiliations:** 1 Department of Human and Social Sciences, University of Bergamo, Bergamo, Italy; 2 Dipartimento di Scienze Umane e Sociali, Università di Bergamo, Piazzale S. Agostino 2, Bergamo, 24129 Italy

**Keywords:** Computerized Language Measures, Multiple Code Theory, Discourse Attribute Analysis Program, Smoothing Operator, Time-DAAP

## Abstract

Bernard Maskit’s work in developing computerized linguistic measures of the referential process has significantly advanced one of the most promising fields of psychotherapy research. His contributions offer the potential for empirical confirmation of the hypotheses contained in Wilma Bucci’s multiple code theory, a general theory of information processing, emotional communication, and psychotherapeutic change. Three key aspects of Maskit’s work yielded rich results for the purposes of both research and clinical practice are highlighted and described: (a) a truly transdisciplinary approach, characterized by the creation of conceptual and operational devices that do not belong to any of the disciplines potentially involved but are instead placed in a third terrain responding to broader, transversal knowledge problems; (b) the ability to develop graphic and formal ways of representing elusive aspects of emotional and linguistic communication, facilitating connections between theory and clinical data, and between clinicians and researchers; and (c) the capacity for a very functional intersubjective connection with Wilma Bucci, his scientific partner and wife, which created a productive new space intertwining psychology and mathematics. Lastly, the future development of Maskit’s work is outlined for the next generation of clinicians and researchers. One of these is the development of the Time-Discourse Attribute Analysis Program, the most current version of the computerized program that applies linguistic measures to texts, which Maskit worked on in his final years. This program must be finalized and validated for its potential heuristic value in both research and clinical practice.

## The Scientific Legacy of Bernard Maskit’s Work for Practice and Research in Psychotherapy

The legacy of human sensibility, intelligence, and honesty that Bernard Maskit left for those who knew him is certainly rich and unforgettable. However, here we would like to focus on some of the many aspects of the scientific legacy he passed on to the broader scientific community, particularly to the group of researchers and clinicians who see Wilma Bucci’s Multiple Code Theory as a fertile ground for scientific and professional development.

## A Truly Transdisciplinary Approach

A key aspect that distinguishes Bernard Maskit’s scientific work on computerized language measures of the Referential Process is its transdisciplinary, rather than merely interdisciplinary, nature. This is a rare occurrence in the sciences. By “transdisciplinary,” we refer to what Piaget (1972) described as the ability to reach a “higher stage of succeeding interdisciplinary relationships which would not only cover interactions or reciprocities between specialized research projects but would place these relationships within a total system without any firm boundaries between disciplines.” In other words, it is the ability to create conceptual and operational devices that do not belong to any of the disciplines potentially involved, but are instead placed in a third terrain to respond to broader, transversal knowledge problems. The technical and mathematical language underlying the linguistic measures of the Referential Process that Maskit developed reflects both an ingenious, out-of-disciplinary-box interpretation of the issues and a broad, in-depth understanding of the mathematical and psychological aspects involved. The mathematical language of these linguistic measures, along with its translation into computer code, goes beyond mere technical and operational solutions– it serves as a way of formalizing and interpreting issues that pertain to the field of human subjectivity in a mathematical way. It is through this higher-level integration of two worlds and modes that new insights and scientific advancements are made. This was certainly the case with Maskit’s work in psycholinguistics as applied to psychotherapy research.

Among the many outcomes of this transdisciplinary approach are several innovative operational solutions (for a more detailed and technical description see Maskit, 2021, 2025; Susskind, 2025), including:

- **The Smoothing Operator**: This sophisticated mathematical procedure was invented to capture the continuous changes over time in the three main functions of the Referential Process—Arousal, Symbolization, and Reorganization. It allows for the reliable, continuous, and accurate calculation and visualization of these functions throughout a psychotherapy session. Simply analyzing the unsmoothed weights of individual words in dictionaries that quantify the presence of the three functions in discourse does not account for, nor does it allow for, a comprehensible visualization of how these functions evolve during a session. Much like how an experienced psychotherapist avoids focusing on individual words or pieces of speech at the expense of understanding their overall clinical meaning, the smoothing operator enables a similar shift from a punctiform, monocular view of the process to a broader, binocular view.
- **The Extraction Process of the Words of the Weighted Dictionaries**: Maskit invented this original procedure to identify the words that best capture and measure a particular function based on independent judges’ multiple ratings of clinical material. This extraction process is the central link between the narrative and the underlying psychological process, achieved through mathematical quantification. It is based on both a technical aspect of estimation and verification of word lists that demonstrates greater reliability and the subjective evaluations of judges of a large sample of extracts from clinical material. We can therefore say that the weighted dictionary extraction consists of creating an *acontextual and general* tool for the reliable estimation of a specific function based on *a contextual and subjective judgement* made by a pool of experts on a sample of text extracts. Once again, it represents a solution that links subjective complexity with its possible formalization and reliable quantification, but without reducing its specificity and complexity.
- **The Covariations Between Linguistic Measures**: These innovative mathematical computations allow for the investigation of joint trends between functions paired in the same time frame—for example, symbolization and reflection during a psychotherapy session. Maskit developed this technique to reveal both expected and unexpected insights from the data by providing a general overview of both functions under analysis, effectively overcoming statistical problems that stem from the non-independence of measurements within the same conversation. This combined vision of several elements offers clinicians a framework to understand a particular clinical phenomenon in a complex, non-simplistic manner. Covariations are an ingenious solution that help in this nuanced investigative process.
- **The Derived Language Measures**: These indices measure the intensity and dominance of a particular function in a given text. The intensity index (Mean High) is the average weight of words in a text that exceed a neutral value; as Maskit metaphorically described, it measures how fast one runs when one is running. It is, thus, an extremely useful measure of the intensity of the speaker’s engagement in the functions activated in their speech. From a clinical point of view, applying the Mean High to the Weighted Referential Activity Dictionary is particularly informative, as it indicates the intensity of the speaker’s emotional involvement when they are emotionally activated in speech. This serves as a measure of emotional regulation, an aspect particularly central to the psychological functioning of many patients. On the other hand, the dominance index (High Proportion) was developed to measure the pervasiveness of a certain function within a text; it represents the percentage portion of the text in which the weights of a particular function associated with words exceed the neutral value. Although this is an apparently simple index, it is capable of detecting the magnitude of the patient’s eventual change in a specific function. For example, in the case of the Weighted Referential Activity Dictionary, it represents the change in translating emotional experience into symbols and narratives, a process that lies at the core of the patient’s psychic change.

## An Embodied Example of a Referential Process

A second aspect worth highlighting is that Bernard Maskit’s work embodied and exemplified the referential process. According to Bucci’s multiple code theory, the referential process connects different systems, languages, and forms of information organization. Specifically, it allows the translation of continuous, analogue modes of experience into representable visual modes, then into symbolic verbal referents, and vice versa. Maskit’s work was an example of this process in several ways: (a) He found a language capable of transforming the elements of continuous experience during speech into images (graphs) and mathematical symbols (indices and associated formulae). In other words, he represented complex psychological processes in space and time, capturing various parts of their complexity in an original and appropriate manner, not only through simple dispersion indices (mean and standard deviation) but also through derived measures, covariations, and smoothed graphs;

(b) He connected the theoretical level of concepts of multiple code theory with elements derived from empirical experience by mathematizing both the theoretical aspects and the elusive complexities of experience. Similar work has been carried out by very few psychologists; one notable example is Matte Blanco (1975), who tried to translate in formal principles some key psychoanalytic concepts but did not combine these formalizations with graphic or empirical instruments that would allow for measurement and visualization.
(c) Maskit found a new mode of communication by developing an accessible and understandable language that allowed the researcher and the clinician to communicate with each other, often enabling each individual to inhabit both roles. Using his computerized language analysis program, a researcher could visually and quantitatively show clinicians trends in specific dimensions of the psychotherapeutic process by applying the computerized measures (and so not subjectively biased) to a whole session, treatment, or parts thereof. At the same time, the clinician can communicate and discuss with the researcher, interpreting the qualitative and quantitative results of the application of the linguistic measures from their subjective point of view. Clinician can also suggest to researcher other dimensions and questions that are important to guiding the research on psychotherapeutic process from within. This creates a circular peer comparison between clinical and research perspectives, enriching both processes of understanding (Jaffe et al., 2024).

**Fig. 1:**
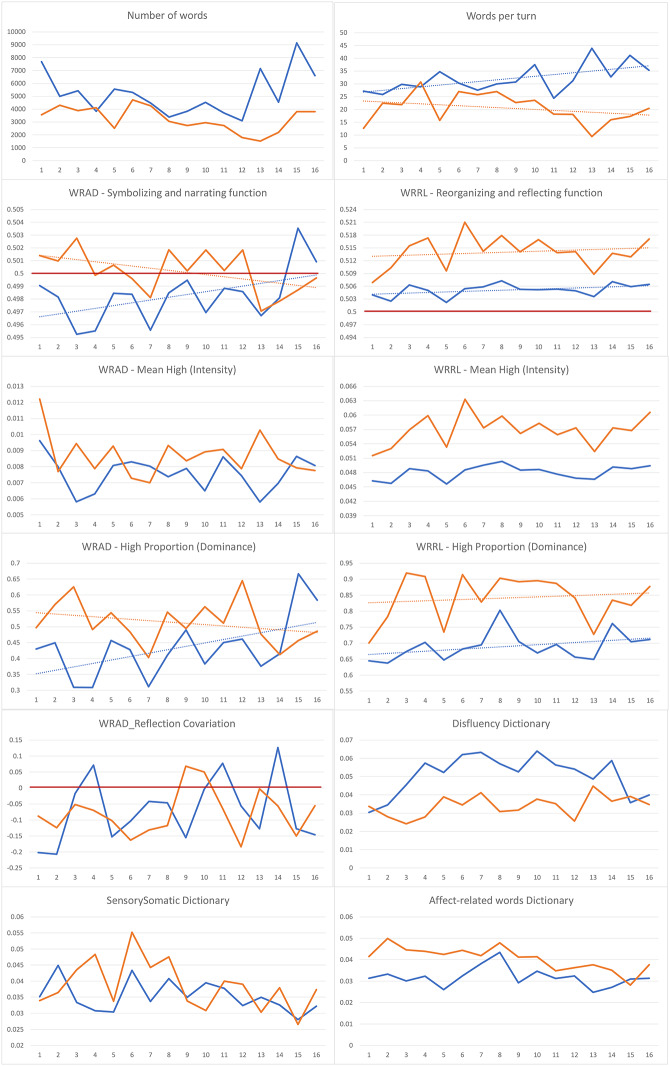
Graphs depicting the development of the main outputs of the application of the computerized linguistic measures of the referential process to the first 4 months of a psychoanalytically oriented therapy Note: The blue line represents the patients’ scores; the orange line represents the therapists’ scores; the dotted line represents the trend of the scores; the red line represents the neutral value

To provide a more concrete and visual example of the points emphasized so far, imagine receiving a set of graphs, like those shown in Fig. 1, from a researcher during treatment. These graphs show a psychoanalytically oriented therapy for a 26-year-old patient with panic attacks that impair his social and work life, conducted at a frequency of one session per week. As the therapist, you would have in your hands a very useful tool for reflecting on some aspects of the ongoing therapeutic process, coming from a third, neutral point of observation– the computer analysis of the type of words used during sessions. Specifically, this analysis would highlight the way the words were used– the linguistic style– indicating the emergence of one of the three main functions of the referential process: arousal (the activation of emotion schemas), symbolization/narration (the verbalization of parts of the emotion schema), and reflection and reorganization (the discovery of personal meanings and connections within narrated material and interpersonal experience). While we do not have the opportunity to delve into the details of each graph here, it can be seen how such graphical representations of the therapeutic process offer a wealth of information about how active each speaker was, the dominance of one of the referential process functions in their speech, how these functions varied over time, and whether they operated in a unified or disjointed manner throughout the treatment.

**Fig. 2:**
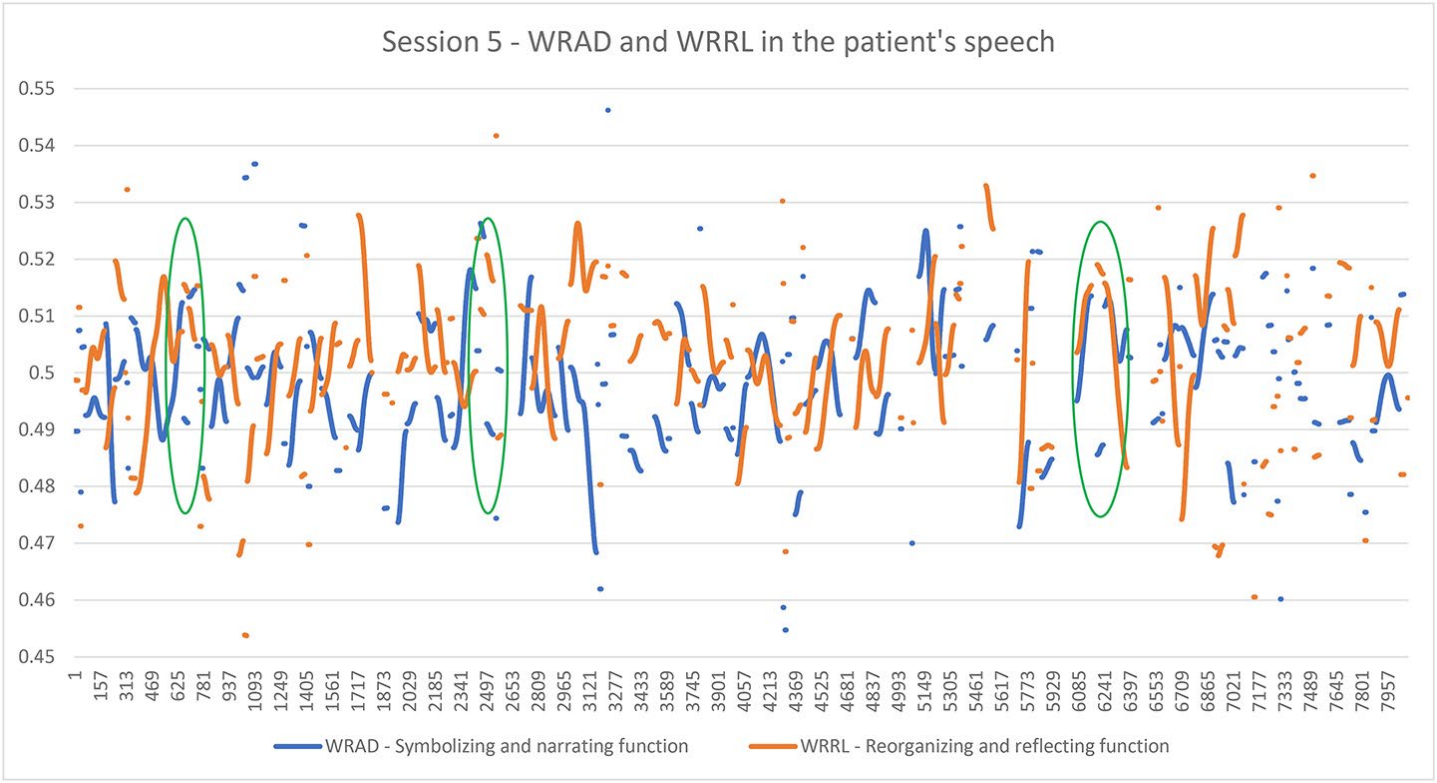
Graphic representation of symbolizing (WRAD) and reorganizing (WRRL) functions during a psychotherapy session Note: The red line represents the neutral value; the first two green ovals indicate points of attention, as a complementary divarication between the functions of symbolization/narration and reorganization/reflection is evident, indicating possible phases of clear reorganization (first oval) and clear symbolization (second oval). The third point of attention (third oval) indicates a clear overlap between the two functions indicating some non-fluidity and functionality of the elaboration process

If we wanted to delve deeper and analyze the micro-process within a specific session (e.g. for supervision purposes), we could examine the graph representing not the entire course of treatment, but the micro-processes of emotional exchange taking place within the session. For example, consider the graph in Fig. 2, which shows the functions of symbolization/narration and reorganization/reflection in the patient’s speech during the fifth session. By examining this graph, we might, for example, want to see which parts of the session correspond to phases where reorganization appears to emerge (see the first green oval, where WRRL increases above the neutral value and then decreases, while WRAD correspondingly decreases below it and then increases). Alternatively, we could focus on phases of narration/symbolization (see the second oval, where WRAD increases above the neutral value and then falls, while WRRL decreases below it and then rises). Even more revealing are the critical points where the two functions overlap, indicating that the process does not follow the usual pattern of the emergence of an emotion, narration, and subsequent reflection. Instead, narration is interspersed with elements of reflection, often serving as a defensive distancing function from emotion (see the third oval in the graph, where WRAD and WRRL rise together above the neutral value).

Such graphs can easily be thought of as analogous to medical tools such as echography, X-ray monitoring, or blood tests. While this analogy partly corresponds to reality, it is also misleading because the processes quantified by the linguistic measures are not objectively captured as those measured and represented by medical tests. Rather, the results from computerized language measures offer clinicians an opportunity to reflect on the complexity of the clinical interaction from a third, neutral point of view. This comparison between internal and external perspectives on the therapeutic process can, in turn, generate clinical and research hypotheses that enrich both the researcher’s and the clinician’s understanding of therapeutic change, both in the specific patient’s treatment and more broadly. This unique vantage on the clinical process has been made possible by Bernard Maskit’s translation of Wilma Bucci’s Multiple Code Theory into formulas, indices, and graphs.

## An Ingenious and Multiplicative Capacity for Intersubjective Connection

A third aspect that added a creative and ingenious quality to Bernard Maskit’s work was the very profound and functional interconnection between him and Wilma Bucci, his scientific partner and wife. This connection was so evident to those who knew them that anyone familiar with Maskit’s work on computerized language measures would see it as intimately connected to Bucci’s multiple code theory, and vice versa. One key to their scientific productivity was undoubtedly their ability to create an effective intersubjective connection of their minds. In terms of multiple code theory, we might describe this as a harmonious communication between their symbolic and subsymbolic systems, which multiplied the potential of both. Those who had the opportunity to see such an intersubjective connection at work often likened their process to the four typical phases of mathematical creativity described by Hadamard (1945)– preparation, incubation, illumination, and verification– revised by Bucci (1997) within her multiple code theory. Here, intuitive, unconscious, and subsymbolic processing combined with systematic, methodical, and symbolic steps. All the creative mathematical solutions, that we only partially mentioned earlier, were created by Bernard in response to requests from Wilma, and in a process of interaction and collaboration with her. They worked side by side, discussing ideas and measures continuously, even during breakfast, lunch, and dinner. Thus, the project of computerized language measures really had two parents. Each contributed something the other lacked, and both were essential to its development. The transdisciplinary approach that lay the proper ground for the progression of Bernard’s work, as previously mentioned, also stemmed from this intersubjective connection between two brilliant minds. Their solutions arose from an in-depth understanding of both the mathematical and psychological sides of the problems they tackled, facilitating a more complex integration, and broadening the possibilities of their understanding.

Wilma Bucci’s insights into the relationship between certain linguistic characteristics of speech and certain underlying psychological functions not only led her to develop the multiple code theory - an innovative theory for understanding emotional information processing and psychotherapeutic change - but also immediately set her in pursuit of empirical evidence to confirm and substantiate her intuitions. It was through her collaboration with Bernard Maskit and his genius that Bucci was able to find a functional modality in this search for a connection between multiple code theory and linguistic analysis. Their process generally worked for them as follows: Wilma would have a psychological vision or identify a need, and Bernard would implement it, often devising new measures that expanded the original vision, sometimes in unforeseen directions. This dynamic and process led to theoretical advancements. For instance, after Bernard created the *Smoothing Operator* solution to generate graphs, he was able to build the derived measures– the Intensity and Dominance indices– tools that had not been anticipated by the original theory. In turn, he saw recognized the need for weighted measures to track other important elements of the process of psychotherapeutic change, such as the Reflecting/Reorganizing and Arousal functions and the emergence of specific emotion schema. Through ongoing scientific conversations with Wilma, Bernard helped develop the psychological frameworks underlying these measures. Moreover, he discovered that the *Smoothing Operator* and the last version of the software he developed to apply the language measure to the texts– the Time-DAAP (discussed later)– enabled his measures to address the effects of word order in human conversation.

## Unlocking and Continuing the Legacy of Bernard Maskit

As with any referential process, the translation of continuous, non-symbolic experience into images and language not only opens up unimagined possibilities but also represents a reduction in the complexity of that experience. However, Bernard Maskit did his utmost to limit the extent of this reduction. One of his tenacious objectives in the final years of his life was to develop an advanced version of the software that applied language measures to text, known as Time-DAAP. This version aimed to add greater complexity to the processes of measurement and graphical representation of the referential process, moving beyond the traditional word-based reference unit of graphs and measurements to a time-based representation of words as they occur in conversation. In future, this could potentially be connected also with elements such as loudness and pitch. For anyone versed in both clinical and research work in psychotherapy, this is certainly a Copernican leap forward. Moving from a succession of words to the time in which the words are told as a basis for measuring and representing psychological processes is like moving from reading the lyrics of a song to listening to them live, from mechanically reading a poem to listening to its author recite it, or from reading the script of an opera to going to the theatre to enjoy it as a live spectator. Unfortunately, Bernard Maskit came only a few steps away from completing this project, which would captured an important additional portion of the complexity of the communication process. It is now up to a new generation of mathematicians, clinicians, and researchers to refine it and unlock its full potential, and to create new creative solutions to capture other aspects of the complexity of emotional communication exchange, such as the influence each speaker has on the next in the referential process (known as the “lead-lag” issue).

Embracing Bernard Maskit’s legacy means adopting the attitudes with which he approached his scientific endeavors: developing a truly transdisciplinary, creative, and complex thinking; addressing questions and problems arising from clinical experience rather than pre-constituted hypotheses; connecting intersubjectively with other minds; maintaining curiosity and irreverence with respect to one’s own established and disciplinary certainties; making coherent choices between theoretical and operational levels; and working with generosity and transparency to deliver theoretical and operational tools to researchers and clinicians that make clinical practice more effective and valuable.
